# Phosphorylation of BCKDK of BCAA catabolism at Y246 by Src promotes metastasis of colorectal cancer

**DOI:** 10.1038/s41388-020-1262-z

**Published:** 2020-04-01

**Authors:** Qin Tian, Ping Yuan, Chuntao Quan, Mingyang Li, Juanjuan Xiao, Lu Zhang, Hui Lu, Tengfei Ma, Ling Zou, Fei Wang, Peipei Xue, Xiaofang Ni, Wei Wang, Lin Liu, Zhe Wang, Feng Zhu, Qiuhong Duan

**Affiliations:** 1 Department of Biochemistry and Molecular Biology, School of Basic Medicine, Huazhong University of Science and Technology, Wuhan, 430030 Hubei China; 2 Tongji School of Pharmacy, Huazhong University of Science and Technology, Wuhan, 430030 Hubei China; 3 State Key Laboratory of Cancer Biology, Department of Pathology, Xijing Hospital and School of Basic Medicine, Fourth Military Medical University, Xi’an, 710032 China; 4 Cancer Research Institute, The Affiliated Hospital of Guilin Medical University, Guilin, 541000 Guangxi China; 5 Key Laboratory of Birth Defects and Reproductive Health of the National Health and Family Planning Commission, Population and Family Planning Science and Technology Research Institute, Chongqing, 400020 China

**Keywords:** Metastasis, Oncogenes, Gynaecological cancer

## Abstract

Branched-chain α-keto acid dehydrogenase kinase (BCKDK), the key enzyme of branched-chain amino acids (BCAAs) metabolism, has been reported to promote colorectal cancer (CRC) tumorigenesis by upregulating the MEK-ERK signaling pathway. However, the profile of BCKDK in metastatic colorectal cancer (mCRC) remains unknown. Here, we report a novel role of BCKDK in mCRC. BCKDK is upregulated in CRC tissues. Increased BCKDK expression was associated with metastasis and poor clinical prognosis in CRC patients. Knockdown of BCKDK decreased CRC cell migration and invasion ex vivo, and lung metastasis in vivo. BCKDK promoted the epithelial mesenchymal transition (EMT) program, by decreasing the expression of E-cadherin, epithelial marker, and increasing the expression of N-cadherin and Vimentin, which are mesenchymal markers. Moreover, BCKDK-knockdown experiments in combination with phosphoproteomics analysis revealed the potent role of BCKDK in modulating multiple signal transduction pathways, including EMT and metastasis. Src phosphorylated BCKDK at the tyrosine 246 (Y246) site in vitro and ex vivo. Knockdown and knockout of Src downregulated the phosphorylation of BCKDK. Importantly, phosphorylation of BCKDK by Src enhanced the activity and stability of BCKDK, thereby promoting the migration, invasion, and EMT of CRC cells. In summary, the identification of BCKDK as a novel prometastatic factor in human CRC will be beneficial for further diagnostic biomarker studies and suggests novel targeting opportunities.

## Introduction

Colorectal cancer (CRC) is one of the most commonly diagnosed malignancies and accounted for 1.8 million new cases and 540 000 deaths worldwide in 2018 [1]. Metastasis accounts for more than 90% of all deaths in patients with various solid tumors, including CRC [2]. Approximately 20% of all patients with CRC present with metastasis at the time of diagnosis and exhibit a poor 5-year survival rate of merely 12.5% [3]. In addition, more than 50% of patients with metastatic colorectal cancer (mCRC) relapse within 5 years after surgery [4]. Therefore, most CRC patients with recurrent distant metastasis are not suitable candidates for conventional therapy [5], and targeted therapy could provide an alternative approach for these patients.

One of the ten characteristics of tumors is the alteration of cell metabolism, which is both a cause and an effect of tumor progression. In the past decade, many enzymes involved in glucose and lipid metabolisms have become the focus of research, including pyruvate kinase M2 (PKM2) [6], hexokinase-2 (HK-2) [7], and isocitrate dehydrogenase (IDH1/2) [8], and all of them have been reported as new targets of tumor-targeted therapy. Amino acid metabolism is also tightly associated with tumor development [9]. Branched-chain amino acids (BCAAs), including leucine, isoleucine, and valine, that are essential amino acids, which are closely related to dysregulated glucose and lipid metabolism, whereas the underlying mechanisms are poorly understood [10].

BCAAs can be employed for protein synthesis or oxidized for energy by tumors [11]. Many cancer types overexpress enzymes that function to degrade BCAAs. Previous studies have shown that the branched-chain aminotransferase 1 and 2 (BCAT1 and BCAT2) enzymes, which catalyze the first step of BCAAs degradation, are overexpressed in gliomas [12], pancreatic cancer [13], and non-small-cell lung carcinoma [14]. Moreover, the isoform BCAT1 has been proposed as a prognostic marker of glioblastoma [15], ovarian cancer [16], breast cancer [17], hepatocellular carcinoma [18], and chronic myelogenous leukemia (CML) [19].

The branched-chain α-keto acid dehydrogenase (BCKDH) complex catalyzes the second rate-limiting irreversible step of BCAAs catabolism [20], and the branched-chain α-keto acid dehydrogenase kinase (BCKDK) phosphorylates and thereby inactivates the E1α (BCKDHA) subunit of this complex to suppress the catabolism of BCAAs [21]. It has been revealed that BCKDK is involved in some hereditary diseases, including Huntington disease [22], autism [23], and metabolic disorders such as obesity [24]. Recently, White et al. reported that inhibition of BCKDK could lower circulating BCAAs, reduce hepatic steatosis, and improve glucose tolerance in Zucker fatty rats [10].

However, the relevance of BCKDK to human cancer remains poorly understood. Therefore, we investigated the role of BCKDK in human cancer and found that elevated BCKDK promoted CRC tumorigenesis by upregulating the MEK-ERK signaling pathway [25]. MEK was indicated to bind with and phosphorylated by BCKDK at the S221 site, thereby activating tumor cell proliferation [25]. Since mCRC was frequently observed in CRC patients, we further investigated the role of BCKDK in mCRC in this study.

The non-receptor protein tyrosine kinase Src, was the first tyrosine kinase found to be significantly more active in the progression of most solid tumors [26]. Src kinase facilitates the activation of various signaling pathways, including the PI3K/Akt pathway, tha β-catenin/E-cadherin complex, and the Ras/MAPK pathway [27]. Furthermore, Src signaling pathways play a crucial role in regulating tumor cell proliferation, survival, tumor angiogenesis [28], epithelial mesenchymal transition (EMT) [29], migration, adhesion, and metastasis [30]. In human CRC, elevated Src activity is considered an independent indicator of poor clinical prognosis in patients [31]. In addition, Src inhibitors have been widely applied in clinical trials [32].

In the present study, we found that BCKDK expression was elevated in mCRC tissues and positively associated with poor survival of CRC patients. However, at the cellular level, direct supplementation with BCAAs failed to facilitate the migration and invasion of CRC cells, indicating that BCKDK exerted its prometastatic function through a BCAAs-independent manner in CRC cells. Using in vitro kinase assays in combination with a publicly available phosphorylation prediction software program, we identified a novel upstream of BCKDK, Src, which phosphorylated BCKDK at the tyrosine 246 (Y246) site in vitro and ex vivo. Importantly, phosphorylation of BCKDK at Y246 by Src enhanced the activity and stability of BCKDK, leading to enhanced migration, invasion, and EMT of CRC cells. Furthermore, elevated p-BCKDK (Y246) expression was also proved to be associated with CRC metastasis and poor prognosis in CRC patients. As an upstream regulator of BCKDK, inhibition of Src downregulated the phosphorylation of BCKDK, suggesting the necessary role of Src in maintaining the prometastatic function of BCKDK. Taken together, these results demonstrate the significance of the Src/BCKDK axis in human CRC and provide a promising novel target for mCRC targeted therapy.

## Results

### BCKDK is upregulated in mCRC and associated with poor prognosis in CRC patients

**Fig. 1:**
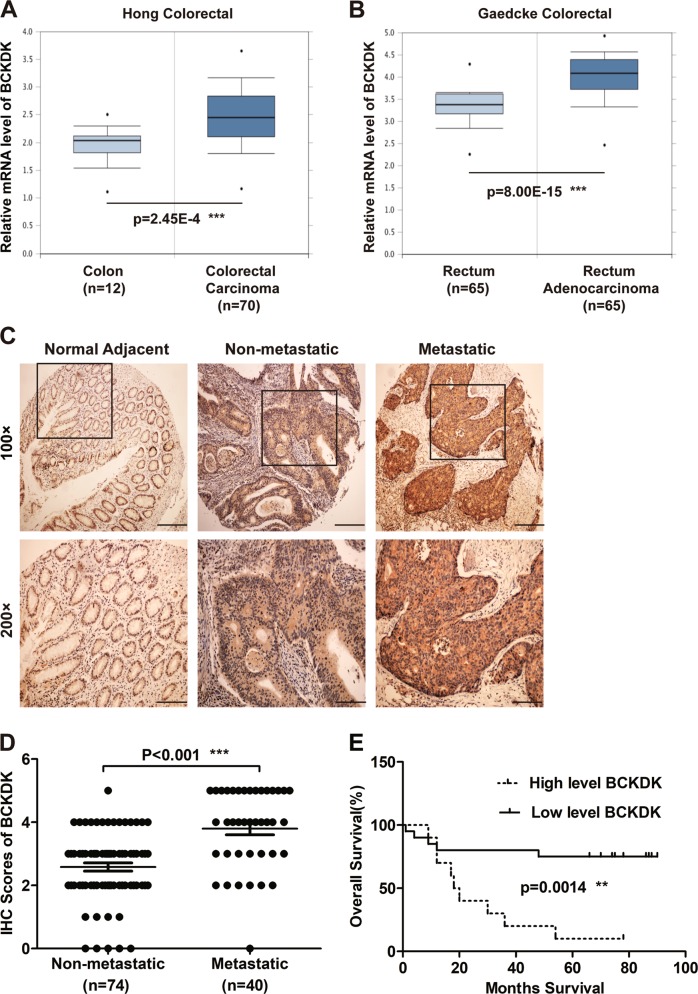
BCKDK is upregulated in mCRC and associated with poor prognosis of CRC patients. BCKDK mRNA expression levels in Hong Colorectal (**a**) and Gaedcke Colorectal (**b**) cell lines. The data comes from the online Oncomine database and are publicly available. The online reporter IDs are 202030_at and A_23_P3823, respectively. **c**, **d** IHC staining of BCKDK in the TMA of CRC. Representative images are shown. (100×, scale bar = 100 µm; 200×, scale bar = 40 µm). **e** Kaplan-Meier analysis of overall survival (OS) in CRC patients. Error bars represent the mean ± SD values. (**p < 0.01, 95% CI = −1.670 to −0.7680).

To understand the role of BCKDK in CRC progression, the mRNA expression level of BCKDK was first analyzed by using the Oncomine database. The online analysis showed that the mRNA level of BCKDK was significantly higher in colorectal carcinoma (Fig. 1a, *n* = 70, ***p < 0.001) and rectal adenocarcinoma (Fig. 1b, *n* = 65, ***p < 0.001) than in corresponding normal tissues.

Next, the protein level of BCKDK was evaluated in the tissue microarrays (TMAs) of CRC patients by immunohistochemical (IHC) staining. The results indicated that BCKDK protein levels were upregulated in metastatic CRC tissues (*n* = 40) compared with nonmetastatic patient tissues (*n* = 74) (Fig. 1c, d, ***p < 0.001). Moreover, the BCKDK protein expression profile and the clinical information of CRC patients were combined to further determine whether BCKDK protein expression was associated with clinical prognosis, and this analysis was achieved by applying the Kaplan-Meier analysis. The outcomes illustrated that CRC patients with high BCKDK protein levels had significantly worse overall survival (OS) than those with low levels (Fig. 1e, **p < 0.01). Based on the above results, we conclude that BCKDK plays an important role in mCRC and might be a valuable indicator for poor prognosis in CRC.

### Knockdown of BCKDK attenuates CRC migration, invasion, and EMT

It has been reported that some metabolites promote cell transformation and metastasis. For example 27-hydroxycholesterol, accelerates the occurrence and metastasis of breast cancer [33]. Previous research has shown that mutations of BCKDH, which catalyzes the irreversible and rate-limiting step of BCAAs catabolism, lead to abnormal accumulation of BCAAs and their metabolites, resulting in maple syrup urine disease. Since BCKDK regulates the activity of the BCKDH complex to influence BCAAs catabolism, which is tightly linked to tumor progression, we wondered whether BCKDK regulated CRC metastasis in a BCAAs-dependent manner. To test this speculation, wound healing cell migration and transwell cell invasion assays were performed to investigate the effects of BCAAs accumulation on the migration and invasion of HCT16 and SW620 cells. The outcomes are shown in Fig. S1A, B, demonstrating that direct supplementation of BCAAs could not accelerate the migration and invasion of CRC cells. These results suggest that BCKDK influences mCRC progression in a BCAAs-independent manner.

**Fig. 2:**
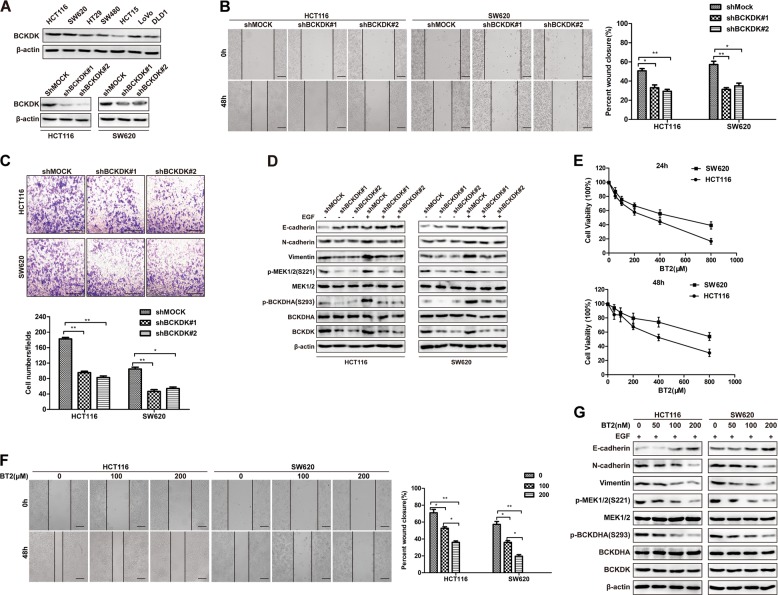
Knockdown of BCKDK attenuates the migration, invasion, and EMT of CRC cells. **a** Expression of BCKDK in seven CRC cell lines (upper panel). BCKDK was knocked down in HCT116 and SW620 cell lines (lower panel). **b** Wound healing cell migration assays of BCKDK knockdown cell lines. Scale bar = 50 µm. **c** Transwell cell invasion assays of BCKDK knockdown cell lines with representative images and quantification shown. The data are presented as the mean ± SD of three replications. *P < 0.05, **P < 0.01. Scale bar = 100 µm. **d** The effects of BCKDK knockdown on the expression of EMT markers were detected by western blot. (EGF: 80 ng/mL, 15 min). **e** HCT116 and SW620 cells were treated with BT2 in the MTT assay. **f** Wound healing assays of cells under BT2 treatment. Scale bar = 50 µm. **g** The effects of BCKDK inhibition induced by BT2 on the expression of EMT markers were detected by western blot (EGF: 80 ng/mL, 15 min).

Further evidences is necessary to define the biological function of BCKDK in mCRC. First, we detected the endogenous expression of BCKDK in seven CRC cell lines (Fig. 2a, upper panel), and BCKDK was knocked down in HCT116 metastatic colorectal carcinoma and SW620 colorectal adenocarcinoma cell lines, which showed high BCKDK expression. We obtained HCT116-shBCKDK(#1/#2) and SW620-shBCKDK(#1/#2) cell lines, as well as corresponding control (shMOCK) cell lines (Fig. 2a, bottom panel). Wound healing assays indicated that BCKDK knockdown inhibited the migration of HCT116 and SW620 cells (Fig. 2b). In addition, transwell assays showed that decreased BCKDK expression impaired the invasive ability of both HCT116 and SW620 cells (Fig. 2c, *p < 0.05, **p < 0.01). As BCKDK is a negative regulator of BCAAs catabolism, knockdown of BCKDK accelerates BCAAs breakdown. Although previous results have demonstrated that high BCAAs stimulation could not accelerate the migration and invasion of CRC cells (Fig. S1A, B), this needs to be confirmed in BCKDK-knockdown CRC cells. Thus, wound healing cell migration and transwell cell invasion assays were performed to investigate the effects of high BCAAs levels on the migration and invasion of BCKDK knockdown CRC cells. The results are shown in Fig. S1C, D. Similarly, direct supplementation with BCAAs could not promote the migration and invasion of BCKDK-knockdown cells. These results further confirm our conclusion that BCKDK exhibits effects on mCRC progression in a BCAAs-independent manner.

The EMT biological program is strongly associated with tumor progression, invasion, and metastasis [34]. EMT is a multistep procedure through which epithelial cells undergo changes in motility and adhesion [35]. Since the EMT process appears to be critical to the metastasis of almost all carcinoma types, including CRC [36, 37], we investigated the effects of BCKDK on the expression of EMT markers (E-cadherin, Vimentin, and N-cadherin). A previous study reported that EGF stimulation could regulate the activity of BCKDK [25]. We performed similar EGF stimulation in BCKDK knockdown cell lines to investigate the mechanism of BCKDK activation to observe whether BCKDK regulates EMT markers under such conditions. MEK1/2 and BCKDHA are known substrates of BCKDK, and p-MEK1/2 (S221) and p-BCKDHA (S293) were detected by western blot to reflect the activity of BCKDK. The results showed that EGF could induce the activity of BCKDK. As the activity of BCKDK increased, EMT strikingly increased (decreased E-cadherin, epithelial marker expression, and increased N-cadherin and Vimentin, mesenchymal marker expressions); Knockdown of BCKDK downregulated its activity, thereby downregulating EMT (Fig. 2d). To further confirm these results, 3,6-dichlorobrenzo(b)thiophene-2-carboxylic acid (BT2), a small-molecule inhibitor of BCKDK [10, 38], was employed to determine the role of BCKDK in CRC. HCT116 and SW620 cells were treated with various concentrations of BT2 for 24 or 48 h to evaluate cell proliferation by MTT assays (Fig. 2e), as described in “Materials and methods”. Similar to BCKDK knockdown, BT2 treatment restrained the migration of HCT116 and SW620 cells at the indicated dose (Fig. 2f). Moreover, western blot analysis showed that BT2 remarkably increased E-cadherin expression and decreased N-cadherin and Vimentin expression in both HCT116 and SW620 cells in a dose-dependent manner (Fig. 2g). Taken together, these results indicate that BCKDK facilitates CRC cell migration, invasion, and EMT.

### Src binds to and phosphorylates BCKDK at the Y246 site in vitro

**Fig. 3:**
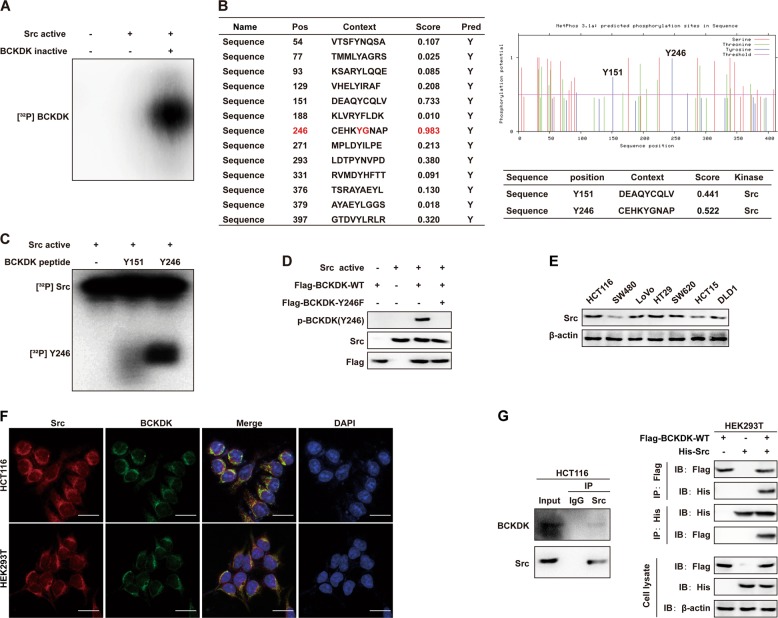
Src binds with and phosphorylates BCKDK at the Y246 site in vitro. **a** Active Src kinase phosphorylated inactive BCKDK substrate in vitro in the presence of [γ-^32^p]ATP as visualized by autoradiography. **b** Potential phosphorylated tyrosine sites (left and upper right panel) and upstream kinase (lower right panel) of BCKDK were predicted by the NetPhos3.1 software program. **c** Active Src phosphorylated BCKDK Y246 peptides in vitro in the presence of [γ-^32^p]ATP. **d** WT and mutated forms of BCKDK protein were used as substrates for active Src in vitro in the presence of ATP. The phosphorylation signal was visualized by western blot with p-BCKDK (Y246) antibody. **e** Expression of Src in seven CRC cell lines. **f** Colocalization of Src and BCKDK in HCT116 and HEK293T cells. **g** BCKDK was coimmunoprecipitated with Src in HCT116 cells (left panel). Exogenous Src coimmunoprecipitated with BCKDK in HEK293T cells (right panel).

The data above indicated that BCKDK has prometastatic potential, and the results were consistent with the positive correlation between BCKDK and metastasis in human CRC specimens (Fig. 1). However, despite the considerable progress in our understanding of the role of BCKDK in CRC, the mechanisms that activate BCKDK during CRC metastasis have not yet been addressed. Protein phosphorylation on tyrosine residues plays a critical role in the regulation of vital processes in cancer progression [39]. The non-receptor tyrosine kinase Src, as a well-established proto-oncogene, was the first tyrosine kinase found to be significantly involved in the progression of almost all kinds of solid tumors, including CRC [26]. Therefore, we wondered whether Src can function as the upstream kinase of BCKDK. To this aim, we first investigated whether Src can directly phosphorylate BCKDK using an in vitro kinase assay. The results indicated that active Src could directly phosphorylate inactive BCKDK in the presence of [γ-^32^P] ATP (Fig. 3a). To clarify the specific phosphorylation sites of BCKDK, we analyzed the protein sequence of BCKDK and found that the phosphorylated conserved sequence pY[A/G/S/T/E/D] of Src [40] was present in the tyrosine 246 site (pY[G]) of BCKDK (Fig. 3b, left panel). In addition, the online NetPhos3.1 software program analysis hinted that there were abundant potential tyrosine phosphorylation sites in BCKDK (Fig. 3b, left panel and upper right panel). The program also implied that Src could be a candidate upstream kinase of BCKDK at the tyrosine 151 (Y151) and tyrosine 246 (Y246) sites (Fig. 3b, lower right panel). Therefore, we obtained two commercially synthesized high-scored BCKDK peptides (Y151 and Y246) (GL Biochem, Shanghai, China) and tested which site could be directly phosphorylated in the presence of [γ-^32^P] ATP in vitro. We observed that, active Src distinctly phosphorylated the Y246 site of BCKDK (Fig. 3c).

To further validate the results of the peptide spectrum, we examined the phosphorylation of full length BCKDK protein by Src using an in vitro kinase assay with phosphospecific antibody recognizing the BCKDK Y246 site, which was prepared commercially, as described in the Materials and Methods. The results of western blot using a p-BCKDK (Y246) antibody indicated that Src could phosphorylate BCKDK-WT, whereas a mutation in BCKDK at Y246 eliminated the phosphorylation (Fig. 3d).

Despite the fact that BCKDK is known to reside in the mitochondrial subcellular compartment, a recent study showed that BCKDK was preferentially localized in the cytosolic fraction, and was also detected in the mitochondrial fraction [10]. Src is well-established as a heterogeneously (both in cytoplasm and nucleus) localized protein [41]. To determine whether there is a possibility of interaction between Src and BCKDK, we first evaluated the expression of Src in seven CRC cell lines (Fig. 3e). Then, an immunofluorescence assay was performed to detect the colocalization of Src and BCKDK in HCT116 and HEK293T cells. The results showed that Src (red) could partially colocalize with BCKDK (green) in the cytosolic fraction (Fig. 3f). Hence, we hypothesized that Src bound to and phosphorylated BCKDK. To verify this hypothesis, co-immunoprecipitation (Co-IP) assay was performed. Endogenous BCKDK could be detected when HCT116 cell lysates were immunoprecipitated with Src antibody (Fig. 3g, left panel). In addition, in the exogenous overexpression Co-IP assay, Src-His and BCKDK-Flag were transfected individually or together, into HEK293T cells. After 48 h, the cell lysates were immunoprecipitated with Flag or His antibodies. Similarly, the results demonstrated that Src co-precipitated with BCKDK (Fig. 3g, right panel). Thus, these findings provide supportive evidence that Src serves as an upstream kinase of BCKDK and phosphorylates BCKDK at the Y246 site in vitro.

### Src phosphorylates and enhances the activity of BCKDK ex vivo

**Fig. 4:**
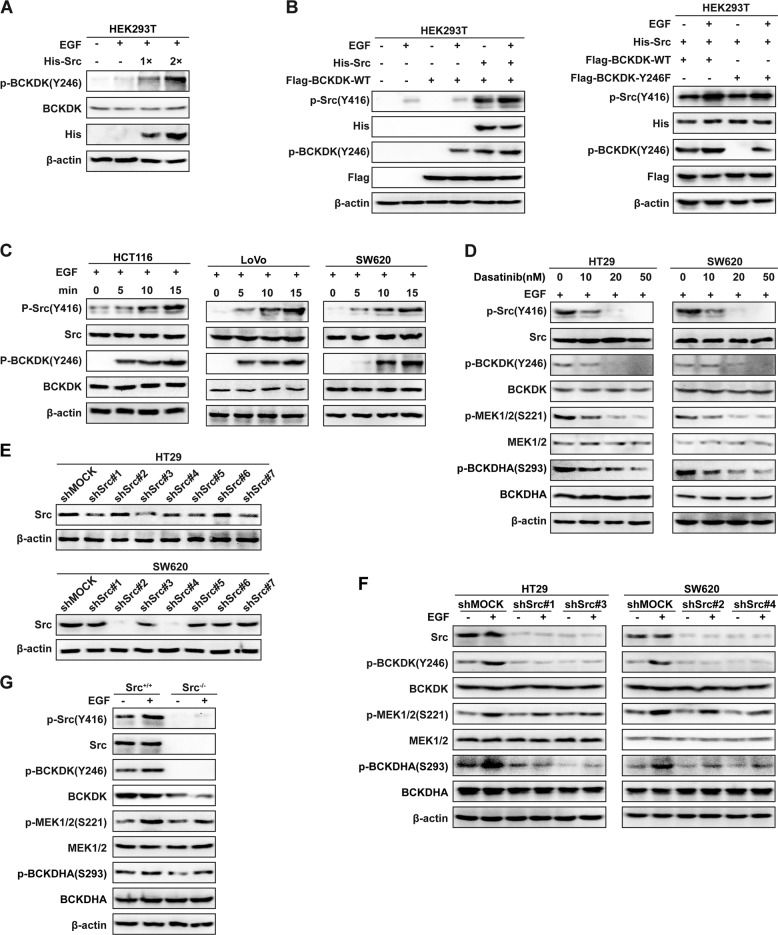
Src phosphorylates and enhances the activity of BCKDK ex vivo. **a** Src phosphorylated BCKDK in a dose-dependent manner under EGF stimulation (EGF: 80 ng/mL, 15 min). **b** Src promoted the phosphorylation of BCKDK in HEK293T cells (left panel). Mutation in BCKDK at Y246 significantly downregulated the phosphorylation effect of Src on BCKDK (right panel). (EGF: 80 ng/mL, 15 min). **c** EGF induced time-dependent phosphorylation of BCKDK in HCT116, LoVo, and SW620 cells. (EGF: 80 ng/mL. 0, 5, 10, and 15 min). **d** HT29 and SW620 cells were treated with dasatinib at the indicated concentrations (0, 10, 20, and 50 nM). (EGF: 80 ng/mL, 15 min). **e** Src was knocked down in HT29 and SW620 cells by nontargeting (shMOCK) shRNA or Src shRNAs (#1-#7). **f** The effects of Src knockdown on the phosphorylation of BCKDK and its downstream targets (MEK1/2 and BCKDHA) were detected by western blot. (EGF: 80 ng/mL, 15 min). **g** Expression of p-BCKDK (Y246), p-MEK1/2 (S221), and p-BCKDHA (S293) was detected in Src^+/+^ and Src^−/−^ cells (EGF: 20 ng/mL, 30 min).

Src is known to be activated in response to numerous growth-stimulating signals, including activation of growth factor receptors, such as EGF receptor [42]. Therefore, we next investigated the regulation of BCKDK phosphorylation by Src under EGF stimulation in cells. As shown in Fig. 4a, increasing amounts of pcDNA_4_-Src-His were transiently transfected into HEK293T cells, and phosphorylation of BCKDK was determined by western blot with the p-BCKDK (Y246) antibody. The results indicated that Src phosphorylated BCKDK in a dose-dependent manner under EGF treatment. Moreover, as shown in Fig. 4b (left panel), when pcDNA_4_-Src-His and pCMV-BCKDK-WT-Flag were individually transfected or co-transfected into HEK293T cells, the phosphorylation of BCKDK at Y246 was dramatically elevated upon Src transfection. In the previous work, we found that the Y246 mutant of BCKDK abrogated the phosphorylation effect of Src on BCKDK in vitro, and we wondered the BCKDK mutant affects the phosphorylation effect of Src on BCKDK ex vivo. Therefore, Flag-BCKDK-WT and Flag-BCKDK-Y246F were co-transfected with His-Src, respectively, into HEK293T cells. Western blot using the p-BCKDK (Y246) antibody was performed to evaluate the phosphorylation effect of Src on BCKDK ex vivo. The results suggested that Src could phosphorylate BCKDK-WT, whereas a mutation in BCKDK at Y246 significantly downregulated the phosphorylation effect (Fig. 4b, right panel).

We confirmed the phosphorylation of BCKDK by Src in HEK293T cells (Fig. 4a, b), but no one has reported the phosphorylation of BCKDK in human CRC cells. Hence, we investigated whether phosphorylation of BCKDK at Y246 could be detected in metastatic HCT116, SW620, and LoVo colorectal adenocarcinoma cell lines. As a result, phosphorylation of Src at Y416 was elevated with increasing EGF exposure time. Consistently, the phosphorylation of BCKDK at Y246 was also increased by EGF stimulation in a time-dependent manner (Fig. 4c). These results suggest that EGF induces Src phosphorylation, which in turn activates BCKDK in HEK293T cells. However, whether BCKDK phosphorylation in CRC cells is induced by Src activation needs further investigation.

To assess the role of Src in BCKDK activation in more detail, phosphorylation of BCKDK and its downstream targets were investigated after Src inhibition. Dasatinib, a small molecule tyrosine kinase inhibitor that targets various tyrosine kinases, especially the Src family [43], was applied to treat colorectal adenocarcinoma HT29 and SW620 cell lines, which exhibited high expression of Src. As shown in Fig. 4d, phosphorylation of Src was inhibited upon dasatinib treatment. Correspondingly, phosphorylation of BCKDK at Y246 decreased in a dose-dependent manner in response to dasatinib, and the expression of p-MEK1/2 (S221) and p-BCKDHA (S293) was also suppressed, while BCKDK activation was reduced. We speculated that Src knockdown could lead to the same effects as dasatinib treatment. To test this hypothesis, we knocked down Src in HT29 and SW620 cells, and the knockdown efficiency was validated by western blot (Fig. 4e). As expected, compared with control cells, HT29 (shSrc#1/#3) and SW620 (shSrc#2/#4) Src knockdown cells had sharply downregulated p-BCKDK (Y246), p-MEK1/2 (S221), and p-BCKDHA (S293) expression (Fig. 4f).

To further identify whether it is possible that loss of Src induces completely inhibits the phosphorylation of BCKDK, Src WT (Src^+/+^) and knockout (Src^−/−^) mouse embryonic fibroblasts (MEFs) cells were used to detect p-BCKDK (Y246) expression by western blot. Indeed, as shown in Fig. 4g, compared with Src^+/+^ cells, we observed absolute inhibition of p-BCKDK (Y246) expression and prominent inhibition of p-MEK1/2 (S221) and p-BCKDHA (S293) expression in Src^−/−^ cells. Altogether, these results indicate that intracellular Src inhibition leads to decreased phosphorylation of BCKDK and its downstream targets. In other words, phosphorylation of BCKDK at Y246 by Src enhances the activity of BCKDK by promoting the phosphorylation of its downstream molecules.

### Src enhances the stability of BCKDK

Interestingly, we observed that the expression of total BCKDK was also sharply decreased in Src^−/−^ cells compared with Src^+/+^ cells (Fig. 4g), suggesting that Src might regulate the stability of the BCKDK protein. To assess whether Src regulates BCKDK protein half-life (*t*_1/2_), Src^+/+^ and Src^−/−^ cells were treated with cycloheximide (CHX) to prevent de novo protein synthesis, and the remaining BCKDK protein level was investigated. Indeed, Src^+/+^ significantly elevated the half-life of BCKDK compared with Src^−/−^ (Fig. S2A). In addition, we transfected HEK293T cells with pCMV empty vector, WT and Y246F forms of BCKDK; after 48 h, the cells were treated with CHX for 0, 3, and 6 h, and the stabilizing effect of Src on BCKDK protein expression in HEK293T cells was analyzed. The results indicated that the half-life of BCKDK-WT was much longer than that of BCKDK-Y246F (Fig. S2B). These results indicated that Src increases the BCKDK protein half-life (*t*_1/2_).

Recently, BCKDK was reported to be ubiquitinated by ubiquitin ligase UBE3B and targeted for degradation [44]. To investigate whether Src enhances the stability of BCKDK by inhibiting its ubiquitination-mediated degradation, Flag-ubiquitin, pcDNA_4_-Src-His, WT, and Y246F forms of BCKDK were transfected into HEK293T cells. After 48 h, the cell lysates were harvested and immunoprecipitated with BCKDK antibody, and the ubiquitination signal was measured by western blot. We found that the presence of Src markedly reduced the ubiquitination of BCKDK-WT, whereas the Y246 mutation resulted in increased ubiquitination of BCKDK (Fig. S2C), suggesting that Src enhances the stability of BCKDK by protecting BCKDK from ubiquitination degradation. Together, these results demonstrate that Src enhances the stability of BCKDK.

### Phosphorylation of BCKDK at Y246 promotes CRC cell metastasis ex vivo

**Fig. 5:**
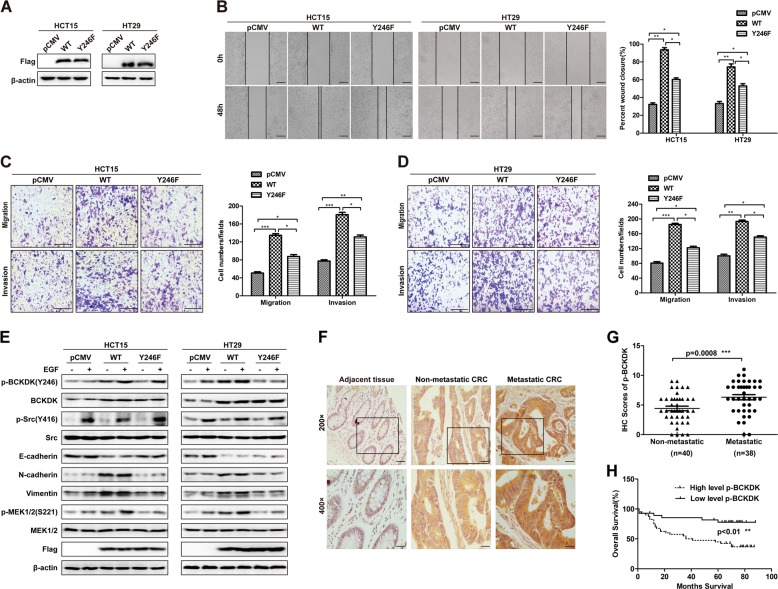
Phosphorylation of BCKDK at Y246 promotes CRC metastasis ex vivo. **a** HCT15 and HT29 cells stably expressing empty vector, WT and Y246F mutant forms of BCKDK were identified by western blot. **b** Wound healing cell migration assays of BCKDK overexpression cell lines. Scale bar = 50 µm. (*p < 0.05, **p < 0.01). Transwell cell migration and invasion assays of BCKDK overexpressing HCT15 (**c**) and HT29 (**d**) cell lines. Scale bar = 100 µm. Representative images of migrated or invaded cells are displayed in the left panels, and the quantification results are shown in the middle and right panels. The data are presented as the mean ± SD of three replications. (*p < 0.05, **p < 0.01, ***p < 0.001). **e** The effects of BCKDK overexpression on the expression of EMT markers in HCT15 and HT29 cells were analyzed by western blot. **f**, **g** P-BCKDK (Y246) IHC analysis in the TMA of CRC. Representative images are shown. (200×, Scale bar = 40 µm; 400×, Scale bar = 20 µm). **h** Overall survival of CRC patients based on IHC scores of p-BCKDK (Y246). The OS curves are built according to the Kaplan-Meier methods (**p < 0.01. 95% CI = −3.063 to −0.7236).

As phosphorylation is one of the most extensively studied posttranslational modifications that orchestrates a variety of cellular functions [39], it is necessary to characterize whether phosphorylation of BCKDK affects the metastasis-related biological functions of BCKDK in CRC cells. To address this issue, we overexpressed BCKDK-WT and BCKDK-Y246F in HCT15 and HT29 cells, which showed low BCKDK expression (Fig. 2a). Cells transfected with pCMV empty vector were used as the negative control. The overexpression efficiency was analyzed by western blot (Fig. 5a). Wound healing assays indicated that overexpression of BCKDK-WT markedly promoted the migration ability of HCT15 and HT29 cells, whereas mutation of BCKDK at Y246 significantly downregulated the promotion effect (Fig. 5b, *p < 0.05, **p < 0.01). Then, transwell cell migration and invasion assays were conducted to elevate the metastatic phenotypes of cells. As shown in Fig. 5c, compared with control and BCKDK-Y246F cells, migration and invasion in BCKDK-WT HCT15 cells were significantly upregulated (*p < 0.05, **p < 0.01, ***p < 0.001). Consistently, in the HT29 cell line, the migration and invasion rates were remarkably upregulated in BCKDK-WT cells compared with control and BCKDK-Y246F cells (Fig. 5d, *p < 0.05, **p < 0.01, ***p < 0.001).

To better understand the effects of BCKDK phosphorylation on CRC cell EMT, we performed western blot analysis. As shown in Fig. 5e, compared with control cells, BCKDK-WT cells exerted a dramatic promotion effect on the expression of N-cadherin and Vimentin. However, BCKDK-Y246F overexpression hardly or moderately upregulated N-cadherin and Vimentin expression in both cell lines. These outcomes indicate that Src phosphorylates BCKDK at Y246 to promote EMT thereby promoting CRC metastasis ex vivo. When combing these results with the results of BCKDK knockdown, we can conclude that EMT-regulated metastasis in CRC cells is coupled not only with the expression of BCKDK itself but also with its phosphorylation status if Src signaling is taken into account.

In the previous work, we demonstrated that BCKDK expression was elevated in mCRC tissues and associated with poor OS in CRC patients (Fig. 1c−e). Therefore, we next determined the level of p-BCKDK (Y246) in CRC patient tissues by IHC staining. As shown in Fig. 5f, g, the expression of p-BCKDK (Y246) was drastically increased in mCRC tissues (*n* = 38) compared with non-mCRC tissues (*n* = 40) (***p < 0.001). Moreover, Kaplan-Meier survival analysis illustrated that elevated p-BCKDK (Y246) expression was associated with bad prognosis in CRC patients (Fig. 5h, **p < 0.01). These demonstrate that p-BCKDK (Y246), as well as BCKDK, is upregulated in mCRC tissues and linked to worse prognosis of CRC patients.

### BCKDK promotes CRC metastasis in vivo

**Fig. 6:**
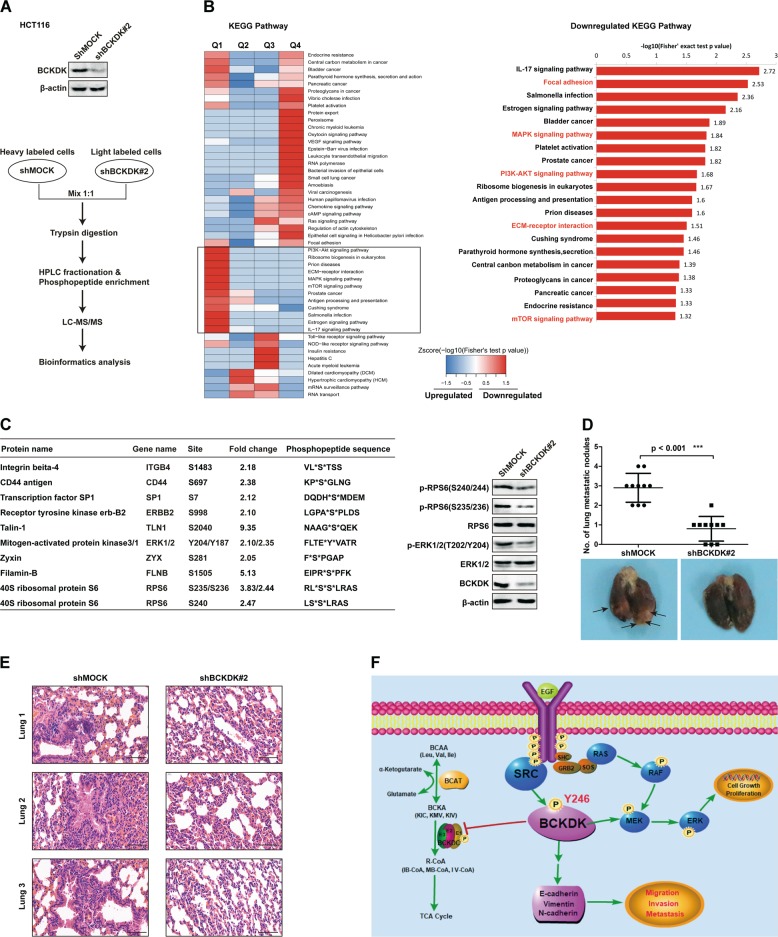
BCKDK promotes CRC metastasis in vivo. **a** Knockdown efficiency of BCKDK in HCT116 cells (upper panel). Systematic workflow of the SILAC-based quantitative phosphoproteomic analyses (lower panel). **b** Functional enrichment-based clustering analysis of the KEGG pathway involved in BCKDK signaling. **c** A list of selected BCKDK-downregulated phosphoproteins (left panel). Western blot of BCKDK knockdown cells (right panel). **d** Knockdown of BCKDK decreased lung metastasis of HCT116 cells in vivo. Error bars represent the mean ± SD, *n* = 10 per group. (***p < 0.001). **e** H&E staining for pulmonary metastatic foci from control and BCKDK-knockdown HCT116 cells. Scale bar = 100 µm. **f** Schematic diagram showing the mechanism of Src/BCKDK/EMT signaling in CRC cells.

To gain comprehensive insight into the BCKDK signaling network, especially signaling involved in cancer cell metastasis, we combined BCKDK knockdown with phosphoproteomics analysis [45]. Control (shMOCK) and BCKDK-knockdown HCT116 cells were used for phosphoproteomic analysis (Fig. 6a, upper panel), and the schematic workflow of stable isotope labeling by amino acids in cell culture (SILAC)-based [46] BCKDK phosphoproteomics analysis is shown (Fig. 6a, bottom panel). We measured the labeling efficiency of cells after six rounds of cell divisions. Then, the effectively labeled samples were analyzed by liquid chromatography-tandem mass spectrometry (LC-MS/MS) that included extensive peptide fractionation and phosphopeptide enrichment. By means of this approach, we built a BCKDK-regulated phosphoproteomic signature. A total of 6 567 phosphosites in 2 815 proteins were identified, and 6 368 phosphosites in 2 748 proteins were quantified (Supplementary Tables S1 and S2).

These phosphoproteins were divided into four groups according to their experiment/control ratio, named Q1 to Q4 : Q1 (0 < ratio ≤ 1/2), Q2 (1/2 < ratio ≤ 1/1.5), Q3 (1.5 < ratio ≤ 2), and Q4 (ratio > 2). With a threshold of fold-change >1, Q1–Q2 and Q3–Q4 were the down- and upregulated groups, respectively. Enrichment-based clustering analyses were performed using the Kyoto Encyclopedia of Genes and Genomes (KEGG) database to profile cellular pathways of Q1–Q4 groups regulated by BCKDK (Fig. 6b, left panel, and Supplementary Table S3). Subsequently, based on a fold-change threshold > 2, Q1 and Q4 groups were significantly down- and upregulated, respectively. We focused on significantly downregulated phosphoproteins. Therefore, KEGG clustering was performed to characterize the alterations in signaling pathways of the Q1 group (Fig. 6b, right panel, and Supplementary Table S4). As shown in the heatmaps, the focal adhesion, MAPK, ECM-receptor, PI3K-AKT and mTOR signaling pathways, and multiple other signaling pathways were downregulated in response to BCKDK-knockdown (Fig. 6b, right panel).

Based on the above KEGG analysis, a selected list of BCKDK-downregulated phosphoproteins is shown in Fig. 6c (left panel): the phosphorylation of multiple molecular factors involved in the EMT program was altered following BCKDK knockdown. As shown, these phosphoproteins belong to various pathways including focal adhesion (ITGB4, ERBB2, ZYX, FLNB, TLN1, and RPS6), ECM-receptor interaction (CD44) and TGF-β (SP1, MAPK1, and MAPK3) signaling. In addition, we performed western blot using existing phospho-antibodies in BCKDK-knockdown cells, and the results indicated that the expression levels of p-RPS6 (S235/236), p-RPS6 (S240/244), and p-ERK1/2 (T202/Y204) were decreased in response to BCKDK-knockdown (Fig. 6c, right panel). These MS data strongly support our previous results that BCKDK regulated EMT in CRC cells. Importantly, our phosphoproteomics approach reveals the powerful potential of BCKDK in regulating multiple cellular responses, including cell proliferation, differentiation, survival, and metastasis.

Finally, lung metastasis mouse models, which were established by tail vein injection of BCKDK knockdown and control (shMOCK) metastatic HCT116 cells, into BALB/C nude mice (*n* = 10 per group), were adopted to validate the significance of BCKDK in CRC metastasis in vivo. As shown in Fig. 6d, injection of BCKDK knockdown cells led to a significantly reduced number of metastatic nodules in the lung compared with that after injection of control cells (***p < 0.001). In addition, hematoxylin-eosin (H&E) staining was used to detect the location of metastatic nodules, and the results indicated that BCKDK knockdown resulted in a lower incidence of lung metastasis in vivo (Fig. 6e). These data from mouse model strongly support the prometastatic function of BCKDK in CRC.

In summary, we report that Src phosphorylates BCKDK at the Y246 site in vitro and ex vivo. Phosphorylation of BCKDK by Src enhances the activity of BCKDK, thus promoting the EMT program to accelerate the metastasis of CRC cells (Fig. 6f).

## Discussion

Previous studies have shown that BCAAs are strongly associated with human metabolic diseases and tumor: elevated plasma BCAAs levels are strongly associated with type 2 diabetes [47] and a high incidence of pancreatic cancer [48]; a BCAAs-rich diet is beneficial for the treatment of hepatocellular carcinoma and liver cirrhosis [49]; and dietary leucine supplements promote the tumorigenesis of pancreatic cancer [50]. However, we found that direct BCAAs accumulation failed to accelerate the migration and invasion of CRC cells ex vivo. Instead, BCKDK, the vital enzyme involved in BCAAs catabolism, was increased in mCRC tissues and associated with poor prognosis of CRC patients. Subsequent studies involving BCKDK knockdown illustrated its biological function in promoting the migration, invasion, and EMT process of CRC cells, as well as the occurrence of distant metastasis in mice. However, the underlying molecular mechanisms of how BCKDK governs the EMT and metastasis of CRC cells are poorly understood at this time.

In 2013, Tonjes et al. observed overexpression of BCAT1, an important enzyme of BCAAs metabolism, in gliomas [12], and its expression in glioblastoma tumors was specific to those carrying IDH1 and IDH2. Mutations in IDH1/2 contribute to a decrease in BCAT1 [12]. Since then, scientists have become interested in this metabolic enzyme and its potential in cancer therapy. Interestingly, in 2015, an inverse relationship between BCAT1 and IDH1/2 was found in epithelial ovarian cancer, where BCAT1 knockdown inhibited the expression of IDH1/2 [16]. Considering their role in TCA cycle, the metabolic link between BCAT1 and IDH1/2 may contribute to elevated cellular migration and invasion in ovarian cancer [16]. Importantly, in 2017, BCAT1 was proved to be highly expressed in CML, and it facilitated cancer progression [19]. All these studies suggest an important role of BCAT1 as a potential prognostic cancer marker. The role of BCAT1 and BCKDK in BCAAs metabolism and the significant status of BCAT1 in tumor progression, raises several questions: Is there any relationship between BCKDK and BCAT1 in CRC development? Does BCKDK promote the metastasis of CRC cells, possibly by regulating BCAT1 activity ? Or does BCAT1 affects the activity of BCKDK in cancer development? Further investigations are needed to address these questions.

The EMT program is controlled by many transcriptional regulators and non-coding RNAs and is associated with malignant behaviors in CRC, including tumor budding, tumor cells dissemination, and drug resistance [36]. An increasing number of findings suggest that tumors undergoing EMT resist conventional drug therapy [36]. Nevertheless, it is unclear how the EMT program impinges on drug resistance, and in comparison with other tumor types, such as breast cancer [51], experimental outcomes on the role of EMT in CRC metastases are still limited [36]. Our data suggest the importance of BCKDK in promoting EMT in the process of CRC metastasis. The SILAC-based BCKDK phosphoproteomics method is well-established as a systematic and unbiased tool for exploring the potential network of BCKDK [46]. Through this approach, we revealed that downregulated BCKDK inactivated the focal adhesion, ECM-receptor interaction and EGFR (MAPK and PI3K-Akt) signaling pathways, all of which are directly or indirectly related to EMT process in cancer progression [36]. However, the concrete regulatory relationship between BCKDK and the LC-MS/MS data shown was not thoroughly investigated in the present study. Therefore, further work should be performed to elucidate the mechanisms by which BCKDK affects EMT-related molecules and to optimize them as new therapeutic agents.

Currently, the recognized targeted therapeutic targets of mCRC are EGFR, VEGF, and VEGFR. Despite the significant advances in drugs approved by the FDA and EMA against these targets, there are still limitations in clinical treatment [52]. Taking Cetuximab as an example, oncogenic activation of EGFR downstream effectors such as KRAS, BRAF, or PIK3CA, leads to sustained activation of the EGFR pathway, which is independent of the state of EGFR itself, and eventually invalidates Cetuximab effects [5, 53]. To address these issues, the clinical value of existing tumor therapeutic targets of mCRC should be seriously evaluated; on the other hand, potential therapeutic targets should be sought to provide novel approaches to overcome the treatment dilemma of mCRC. Our results showed that elevated BCKDK and p-BCKDK (Y246) expression levels were positively associated with mCRC and negative prognosis in CRC patients, suggesting its potential role as a therapeutic target in mCRC. Hence, it is necessary to conduct more basic experiments and expand CRC pathological specimens to further confirm the impact of BCKDK in mCRC, thereby providing more reliable and comprehensive conclusions for clinical trials.

Src is well-established oncogene. Indeed, increased Src activity has been linked to the progression and malignancy of colorectal cancer, breast cancer, ovarian cancer, and lung carcinomas [54], as well as poor clinical prognosis and resistance to cancer therapy [55]. Notably, in this study, Src was proved to be a novel positive regulator of BCKDK activity, which contributes to the metastasis of CRC. Inhibition of Src suppressed the activity and ability of BCKDK. Importantly, we first generated a p-BCKDK (Y246) antibody, and p-BCKDK (Y246) expression was detected in vitro and ex vivo, and by IHC staining analysis. However, whether the p-BCKDK (Y246) antibody has similar efficiency in other cancer types and patient tissues requires further study. Briefly, in human CRC, our findings provide novel insight into the option of promising double-target (targeting both Src and BCKDK) therapy for mCRC.

## Materials and methods

### Cell culture and transfection

The HCT116, SW620, DLD1, HCT15, SW480, HT29, and HEK293T cell lines were purchased from American Type Culture Collection (ATCC; Manassas, VA, USA). The CRC cell lines HCT116, SW620, HCT15, HT29, SW480, and DLD1 were grown in RPMI 1640 Medium (Gibco, Grand Island, NY) with 10% fetal bovine serum (FBS; Gibco), and HEK293T cells were cultured in Dulbecco’s modified Eagles’ medium (DMEM, Gibco, Grand Island, NY) supplemented with 10% FBS. Colorectal adenocarcinoma LoVo cell line was obtained from the Cell Bank of the Chinese Academy of Sciences (Shanghai, China) and were cultured with F-12K medium (Sigma, St. Louis, MO) containing 10% FBS. The Src^+/+^ and Src^−/−^ MEFs cells were gifts from Imamoto A (University of Chicago, Chicago, IL 60637, USA) and were cultured in Dulbecco’s modified Eagles’ medium (DMEM, Gibco, Grand Island, NY) with 10% FBS. All cells applied in this study were cultured at 37 °C in a humidified 5% CO_2_ atmosphere. The cell transfection assays were performed using Simple-fect reagent (Signaling Dawn Biotech, Wuhan) following the manufacturer’s instructions. Corresponding blank vectors were added for adjustment to ensure that an equal amount of total DNA was transfected for each individual group. Puromycin and G418 (Sigma, St. Louis, MO) were applied for knockdown and overexpression stable cell lines screening, respectively.

### Antibodies and regents

The β-actin (1:1000, sc-130656) and BCKDK (E-12) (1:1000, sc-374425) antibodies were purchased from Santa Cruz Technology, Inc. (Santa Cruz, CA, USA). Flag antibody (1:1000, #F1804, #F7425) was purchased from Sigma-Aldrich (St. Louis, MO, USA). His Mouse monoclonal antibody (1:1000, 4E6) was obtained from Pregene, Inc (Beijing, China). All the following antibodies were purchased from Cell Signaling Technology (Danvers, MA, USA): p-BCKDHA (S293) (1:500, #40368), BCKDHA (1:500, #90198), MEK1/2 (1:1000, #8727), p-MEK1/2 (S221) (1:1000, #2338), p-ERK1/2 (T202/Y204) (1:2000, #4370), ERK1/2 (1:2000, #4695), p-RPS6 (S235/236) (1:1000, #2211), p-RPS6 (S240/244) (1:1000, #2215), RPS6(1:1000, #2217), E-cadherin (1:500, #3195), N-cadherin (1:500, #13116), and Vimentin (1:500, #5741). p-BCKDK (Y246) antibody was prepared by Abiocode, Inc (shanghai, China). Rabbit (1:3000, #E030120) and mouse (1:3000, #E030110) antibodies were obtained from EarthOx Life Sciences (San Francisco, CA, USA). We purchased BT2 (Cat No. ZC-26488) from ZZBIO. CO.LTD (Shang Hai, China). Dasatinib (Cat No. S1021) was purchased from Selleckchem, Inc (Houston, TX, USA).

### Plasmids and shRNAs

The plasmids of pCMV-BCKDK-WT-Flag, pCMV-BCKDK-Y246F-Flag, pLKO.1-shSrc (#1-#7) and pLKO.1-shBCKDK (#1-#2) were constructed by our laboratory. All PCR products were confirmed by sequencing analysis (Sangon Biotech, Shanghai). The sequences for shRNAs against human BCKDK are: 1. 5′-CCGGTCAGGACCCATGCACGGCTTTCTCGAGAAAGCCGTGCATGGGTCCTGATTTTTG-3′; 2. 5′-CCGGACGCTGACTTCGAGGCTTGGACTCGAGTCCAAGCCTCGAAGTCAGCGTTTTTTG-3′. The sequences for shRNAs against Src are: 1. 5′-CCGGGCTCGGCTCATTGAAGACAATCTCGAGATTGTCTTCAATGAGCCGAGCTTTTTG-3′; 2. 5′-CCGGGACAGACCTGTCCTTCAAGAACTCGAGTTCTTGAAGGACAGGTCTGTCTTTTTG-3′; 3. 5′-CCGGGTCATGAAGAAGCTGAGGCATCTCGAGATGCCTCAGCTTCTTCATGACTTTTTG-3′; 4. 5′-CCGGTCAGAGCGGTTACTGCTCAATCTCGAGATTGAGCAGTAACCGCTCTGATTTTTG-3′; 5. 5′-CCGGGCTGACAGTTTGTGGCATCTTCTCGAGAAGATGCCACAAACTGTCAGCTTTTTTG-3′; 6. 5′-CCGGCATCCTCAGGAACCAACAATTCTCGAGAATTGTTGGTTCCTGAGGATGTTTTTTG-3′; 7. 5′-CCGGCATCCTCAGGAACCAACAATTCTCGAGAATTGTTGGTTCCTGAGGATGTTTTTG-3′. A scrambled siRNA with a sequence lacking significant homology to the human genome database was used as the control siRNA (shMOCK).

### Western blot and immunoprecipitation (IP)

Cells cultured for western blot were harvested in RIPA buffer (0.5% sodium deoxycholate, 1× PBS, 1 mmol/L Na_3_VO_4_, 1% NP-40, 0.1% SDS, 1 mmol/L aprotinin, and 1 mmol/L PMSF), sonicated three times and centrifuged to obtain the supernatant. For co-IP assays, IP buffer (50 mM tris-HCl pH 7.4, 1% NP40, 150 mM NaCl, 1 mM DTT, and 1 mM EDTA) was used to acquire IP samples. After sonicated and centrifuged, the supernatant fractions containing equal amounts of protein were subjected to incubation with corresponding antibody and protein A/G plus-agarose beads (Santa Cruz Biotechnology, Inc.). For western blot, protein samples were boiled and separated on 7.5–15% SDS-PAGE gels followed by transferring to PVDF membranes (Millipore, Billerica, MA, USA). After blocking with 5% nonfat milk in Tris-buffered saline containing 0.1% Tween-20 for 30 min, the membranes were incubated with specific primary antibodies overnight at 4 °C. Finally, antibody-bound proteins were detected by chemiluminescence (BIORAD, USA).

### Phosphoproteomic analysis

For Stable Isotope Labeling by Amino Acids in Cell Culture (SILAC)-based phosphoproteome experiments, HCT116 cells were infected with lentiviruses encoding control hairpin (shMOCK) or shRNA specific for BCKDK (shBCKDK#2), then BCKDK knockdown efficiency was tested by Western blot before proceeding to phosphoproteomics experiments. Subsequently, cells were metabolically labeled with the SILAC protein quantitation kit (Pierce, Thermo): the control cells were labeled with “heavy isotopic amino acids” (L-^13^C_6_-Lysine/L-^13^C_6_^15^N_4_-Arginine) and shBCKDK#2 cells were labeled with “light isotopic amino acids” (L-Lysine/L-Arginine). To achieve more than 98% labeling efficiency, the cells was cultured for more than six generations before being harvested. First, the labeling efficiency was detected. Next, a total of 2 mg of mixed cell lysates (1:1, 1 mg of control cells and 1 mg of shBCKDK#2 cells) were digested with trypsin. The tryptic peptides were fractionated by high pH reverse-phase HPLC using Agilent 300Extend C18 column (5 μm particles, 4.6 mm ID, 250 mm length). Then each fraction was subjected to phosphopeptide enrichment. The specific peptides were selected for in-solution digestion and liquid chromatography-tandem mass spectrometry (LC-MS/MS) using NCE setting as 28 and the fragments were detected in the Orbitrap at a resolution of 17,500. The resulting MS data were processed using Maxquant search engine (v.1.5.2.8). Finally, bioinformatics methods analysis were performed to analysis the MS data.

### Wound healing cell migration assay

The wound healing assays were applied to determine the migration ability of cells. 2 × 10^5^ cells were cultured in a six-well plate until 80–90% confluence and then carefully scratched with a 10 μL pipette tip. After washing three times with 1 × PBS to remove detached cells, images in 10 different wound fields were captured at respective time points (0 and 48 h) to evaluate the migration of cells.

### Transwell assay (cell migration and invasion assay)

Chambers (#3422, 8 µm pore, Corning, NY, USA) without or with matrigel (#356234, BD Biosciences, CA, USA) were used to investigate migration and invasion ability of cells, respectively. Systems without Matrigel were used to measure the migration ability of cells. 1 × 10^5^ cells suspended in 150 mL serum-free medium were seeded onto the upper chamber of 24-well plates, and 700 mL of medium with 10% FBS was added to the lower chamber. 48 h later, the medium was removed from the upper chamber. The non-invading cells on the upper side of the chamber were removed thoroughly with a clean cotton swab. Then cells on the bottom side of the membrane were fixed with 4% paraformaldehyde for 30 min, the migrated cells were stained with 0.1% of crystal violet (Sangon Biotech) for 15 min Finally, the stained cells were counted by microscopy. Results represent the average number of cells in three fields per membrane in triplicate inserts. As for cell invasion assays, the matrigel was diluted according to the manufacturer’s recommendations and added onto the chambers before seeding cells, then performed in the same manner as cell migration assays.

All the experiments above were performed in triplicate, and results were presented as mean value ± SD.

### MTT assay

The cell viability was analyzed by MTT assay. HCT116 and SW620 cells (1 × 10^4^/well) were seeded in 96-well plates for 24 h, then treated with BT2 (0, 100, and 200 μM) for 24 and 48 h, respectively. Subsequently, 20 μL 0.5% MTT was added per well and incubated for 4 h. The culture media was discarded and 150 μL of dimethyl sulfoxide (DMSO) was added. After 15 min, when the crystal was fully dissolved, the absorbance value was measured at OD490 nm on an enzyme immunoassay analyzer (Bio-Rad).

### In vitro kinase assay

The inactive human BCKDK recombinant protein (# TP303601) was obtained from Beijing OriGene Technology. Src active kinase was purchased from Millipore Corp (Billerica, MA, USA). The inactive substrate (BCKDK) and the active kinase (Src) were incubated in a 30 μL reaction system (1 × kinase buffer containing 1 μCi [γ-^32^P] ATP) at 37 °C for 2 h . The reactions were stopped by adding 5× SDS loading buffer. Finally, the phosphorylation signal was detected by autoradiography (Amersham Typhoon IP. GE, USA).

As for in vitro kinase assay in Fig. 3d, We obtained pCMV-BCKDK-Y246F-Flag mutant plasmid based on the pCMV-BCKDK-WT-Flag plasmid. HEK293T cells were transiently transfected with the pCMV-BCKDK-WT-Flag and pCMV-BCKDK-Y246F-Flag. 48 h later, the cell lysates were collected and immunoprecipitated with Flag antibody, then the immunoprecipitate were incubated with active Src kinase in the presence of 100 μmol/L ATP at 37 °C for 2 h. The phosphorylation was detected by western blot using p-BCKDK (Y246) antibody.

### Immunofluorescence assay

The HCT116 and HEK293T cells seeded on a coverslip (Thermo Fisher Scientific, Waltham, MA, USA) were fixed in 4% paraformaldehyde for 20 min, permeabilized with 0.2% Triton X-100 for 30 min, and blocked in 10% FBS for 30 min Then the cells were incubated overnight with primary antibodies (Src and BCKDK, both diluted 1:50) at 4 °C. On the second day, the cells were incubated for 2 h at room temperature with the Alexa Fluor 488 (green for BCKDK) or Alexa Fluor 546 (red for Src) secondary antibodies while keeping in the dark. The chromatin was stained by DAPI. Images were captured by LSM700 confocal microscopy (Zeiss, Oberkochen, Germany).

### Patients samples and immunohistochemistry (IHC)

TMAs of human CRC were obtained from Urology, Xijing hospital of the Fourth Military Medical University. This study was performed with the permission of the ethical committee of the Fourth Military Medical University. The IHC staining analysis was performed using BCKDK (1:50) and p-BCKDK (Y246) (1:50) antibodies. The immunostaining intensity were graded following the Remmele scoring method. The positive group was defined by score > 3. Representative images were obtained by magnified (×100, ×200, and ×400) with an Olympus Imaging System Microscope.

### CHX treatment

The MEFs cells (Src^+/+^ and Src^−/−^) and transiently transfected HEK293T cells were treated with 20 and 80 ng/mL EGF, respectively. Then 100 μg/mL of cycloheximide (CHX, Sigma, St. Louis, USA) was supplemented to the medium to block the new protein synthesis. Cells were harvested at a range of time points and the levels of aim proteins were analyzed by western blot.

### Xenograft metastasis model and H&E

The BALB/C-nu/nu nude mice were obtained from Beijing Vital River Laboratory Animal Technology Corp. For in vivo tumor metastasis assays, 5–6-week-old BALB/C-nu/nu nude mice (*n* = 10 per group) were injected in their tail veins with 1 × 10^6^ control (shMOCK) cells or BCKDK knockdown (shBCKDK#2) cells. The mice were observed daily and sacrificed after 8 weeks. The number of tumor nodules on the surface of lungs was counted and statistically evaluated. The collected lungs were fixed with formalin and submitted for hematoxylin-eosin staining (H&E). Medical Ethics Committee of the Tongji Medical College, Huazhong University of Science and Technology approved the protocols for animal experiments.

### Statistical analysis

Statistical analysis in this study were performed using *GraphPad Prism 5.0* Software (GraphPad, San Diego, CA). All data were collected from three independent experiments and the results were expressed as mean ± SD. 1-way ANOVA or a two-tailed Student’s *t* test was performed to analyze the statistically significance. *p* values < 0.05 was considered as significant. *p < 0.05; **p < 0 01; ***p < 0.001.

## Supplementary information

Supplementary figure and table legends

Figure S1

Figure S2

Supplementary Table S1

Supplementary Table S2

Supplementary Table S3

Supplementary Table S4
