# Supplementary material for: Phosphorylation of BCKDK of BCAA catabolism at Y246 by Src promotes metastasis of colorectal cancer
Source: Oncogene. 2020 Apr 1;39(20):3980–96. doi: 10.1038/s41388-020-1262-z (PMC7220852; doi:10.1038/s41388-020-1262-z)
Supplement: Supplementary file 1 — Supplementary figure and table legends [file 41388_2020_1262_MOESM1_ESM.docx]

**Figure S1. BCAAs accumulation fails to promote the migration and invasion of CRC cells.**

**A,** Wound healing cell migration assays of HCT116 and SW620 cells. Scale bar = 50 µm ns: not significant. **B,** Transwell cell invasion assays of HCT116 and SW620 cells. BCAAs: 0, 1200 and 2400 µM. Scale bar = 50 µm, ns: not significant. **C,** Wound healing cell migration assays of BCKDK knockdown CRC cells. BCAAs: 2400 µM. Scale bar = 50 µm, ns: not significant. *, *P* < 0.05, **, *P* < 0.01. **D,** Transwell cell invasion assays of BCKDK knockdown CRC cells. BCAAs: 2400 µM. Scale bar = 50 µm, ns: no significant. *, *P* < 0.05, **, *P* < 0.01.

**Figure S2. Src enhances the stability of BCKDK.**

**A**, Src^+/+^ and Src^-/-^ cells were stimulated by EGF (20 ng/ml, 30 min) followed by CHX (100 μg/ml) treatment. **B**, HEK293T cells were transiently transfected with empty vector, WT and Y246F mutated forms of BCKDK. Transfected cells were stimulated by EGF (80 ng/ml, 15 min) followed by CHX (100 μg/ml) treatment. **C,** PcDNA_4_-Src-His, BCKDK-WT-Flag and BCKDK-Y246-Flag were co-transfected with Flag-ubiquitin, and after 48 h, the cell samples were immunoprecipitated with BCKDK antibody. Then, the immunoprecipitates were detected with ubiquitin antibody by western blot. The transfection efficiency and equal protein loading were verified by western blot using whole cell lysates.

**Table S1. LC-MS/MS spectrum database search analysis summary.**

**Table S2. All BCKDK-related phosphoproteins.**

**Table S3. KEGG pathways of all (Q1-Q4) groups.**

**Table S4. Significantly downregulated KEGG pathways.**
