# Supplementary figures and images for: Phosphorylation of BCKDK of BCAA catabolism at Y246 by Src promotes metastasis of colorectal cancer
Source: Oncogene. 2020 Apr 1;39(20):3980–96. doi: 10.1038/s41388-020-1262-z (PMC7220852; doi:10.1038/s41388-020-1262-z)

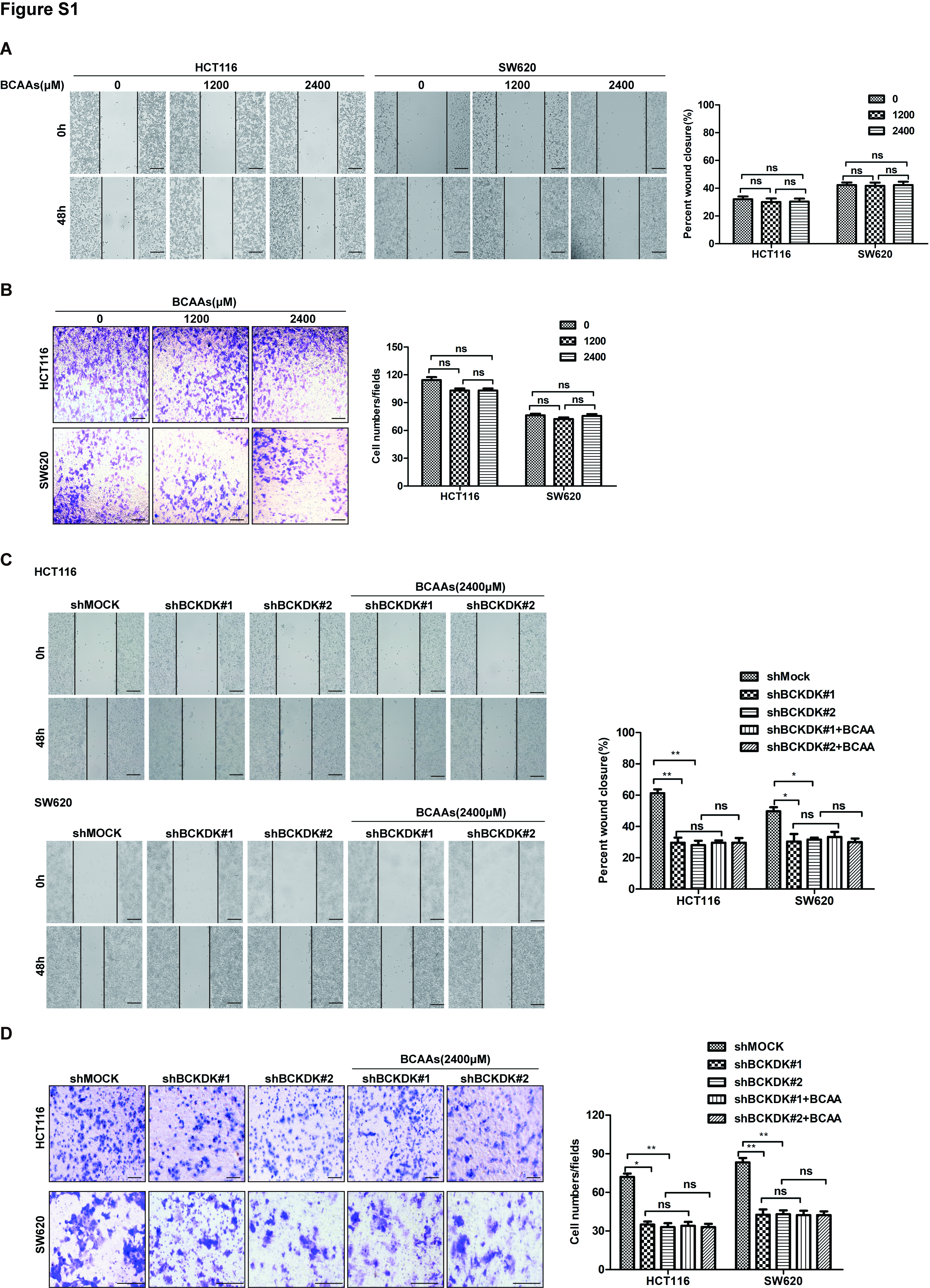

Supplement: Supplementary file 2 — Figure S1 [file 41388_2020_1262_MOESM2_ESM.tif]

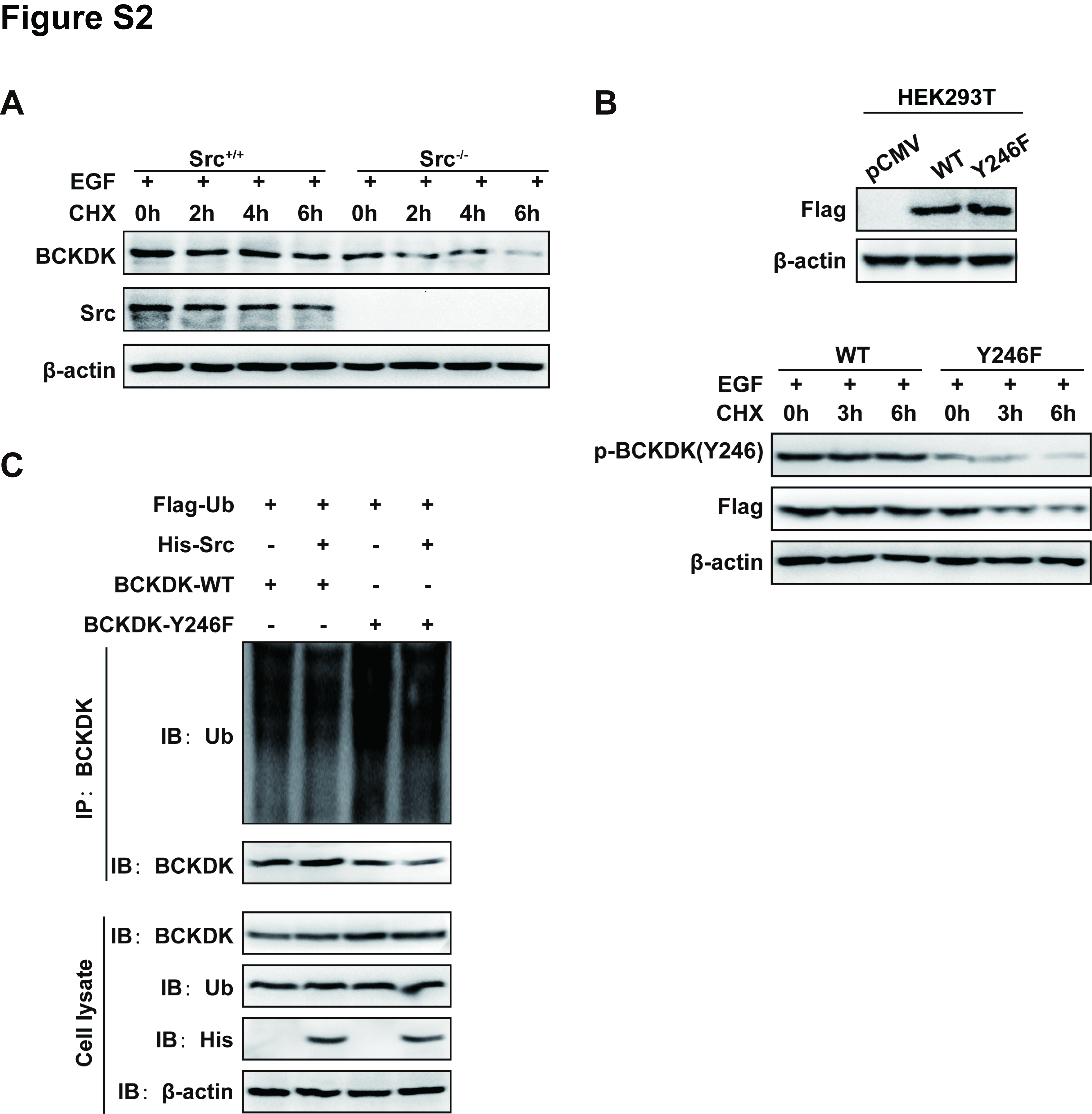

Supplement: Supplementary file 3 — Figure S2 [file 41388_2020_1262_MOESM3_ESM.tif]
